# Real Response to Therapy in Chronic Hepatitis C Virus Patients: A Study From Iran

**DOI:** 10.5812/hepatmon.6151

**Published:** 2012-09-15

**Authors:** Najmeh Namazee, Shahnaz Sali, Sorour Asadi, Mostafa Shafiei, Bita Behnava, Seyed Moayed Alavian

**Affiliations:** 1 Infectious Disease and Tropical Medicine Research Center, Shahid Beheshti University of Sciences, Tehran, IR Iran; 2 Research Center for Gastroenterology and Liver Diseases, Baqiatallah University of Sciences, Tehran, IR Iran

**Keywords:** Hepatitis C, Chronic, Peginterferon Alfa-2a, Ribavirin

## Abstract

**Background:**

Despite significant advances in the treatment of chronic hepatitis C in the past decades, factors which can affect response rates to combination therapy; peginterferon and ribavirin, are still under study and reaching sustained virological response (SVR) is affected by several different factors.

**Objectives:**

To investigate predictor factors contributing to SVR in Iranian patients.

**Patients and Methods:**

The present non-randomized, clinical trial was conducted on 100 patients referred to the Tehran Hepatitis Center in 2009-2011. The patients were administered combined peginterferon α-2a-ribavirin treatment, based on the standard protocol of the Iranian Ministry of Health. At the end of the treatment, the SVR rate and predictors were evaluated.

**Results:**

The mean age of the patients was 42 and 78% were male. Genotype 1a was the most common (70%) and 55% of patients were treatment naïve. The outcomes showed that 12%, 16% and 22% patients were; non-responders, breakthroughs and relapsers, respectively, while 50% of the patients reached SVR. Patients reaching SVR were aged 40 years or lower, they were less likely to have been a non-responder in prior treatments, more likely to have a non-1a genotype and a higher number had an HCV RNA of less than 600 000 IU/ml. The multivariate analysis showed that an age of 40 or lower (OR = 3.74, CI95% = 1.52-9.22), a non-1a genotype (OR = 3.71, CI 95% = 1.40-9.81) and an HCV RNA less than 600 000 IU/ml (OR = 2.52, CI 95% = 1.03-6.15) may be useful SVR predictors.

**Conclusions:**

The findings of the present study showed that half of the patients reached SVR through combined peginterferon α-2a and ribavirin treatment, the majority of whom had genotype 3a and a minority had genotype 1a. In addition, an age of 40 or lower, non-1a genotype and a viral load less than 600 000 IU/ml were strong SVR predictors.

## 1. Background

The treatment regimen of chronic hepatitis C has changed significantly over the past decades in antiviral treatment of hepatitis C virus (HCV) infection and combination therapy of peginterferon-ribavirin has become a standard around the world (1) and in Iran, too (2). The efficacy of antiviral treatment of HCV is measured through sustained virological response (SVR), which is operationally defined as the absence of HCV RNA (detectable through RT-PCR) within six months of treatment termination (3). Reaching SVR not only prevents advancement towards cirrhosis, HCC and liver failure, but it may also improve a patient’s quality of life (4-6). In addition, the majority of affected patients remain in blood and liver remission with long-term effects of 99-100% (7, 8). Although combination therapy of pegylated interferon and ribavirin significantly increases SVR and increases the probability of reaching SVR by 50% (9), the degree of response depends on a variety of factors and these may also differ in various patient populations (10). Viral genotype, viral load, patient age, BMI, race, environment and several other factors have been shown to correlate with SVR (11-16). Prolonged antiviral treatment with numerous complications and medical costs on the one hand and the multiplicity of factors affecting response to treatment on the other hand, warrant the identification of the predictive variables of reaching SVR as a leading morbidity prevention factor in selected populations (17). Although a few studies on the effects of this therapy on HCV have already been conducted in Iran, they have used either a small sample size or have not considered predictor variables (18-23). For instance, Alavian et al., studied SVR predictor variables, but their research only included hemophilic patients (12).

## 2. Objectives

Therefore, the present study attempts to study a range of predictors for reaching SVR including; patient characteristics, viral characteristics and the results of tests such as endof- treatment leukopenia, neutropenia, thrombocytopenia and aspartate aminotransferase (AST) to platelet ratio index or APRI, in chronic hepatitis C patients receiving combination therapy of peginterferon α-2a and ribavirin.

## 3. Patients and Methods

### 3.1. Study Design and Patient Selection

The present non-randomized, open-label clinical trial was conducted in the 2009-2011 period. A sample of 100 HCV patients with a minimum age of 15 was selected from the patients referred to the Tehran Hepatitis Center. This center is a referral center which receives patients from all parts of Iran. The selected patients had chronic hepatitis C, evidenced by a liver biopsy performed not more than one year before the study screening. They had a HCV RNA level higher than 50U/ml six months before the study and they were not under treatment. Other entry criteria included an Hb > 12g/dl, an absolute neutrophil count (ANC) > 1500/mm^3^ and blood platelets higher than 80 000/mm^3^.

Exclusion criteria included simultaneous infection with hepatitis B or human immunodeficiency virus (HIV), active liver disease, existence of liver disease with a cause other than hepatitis C, hepatocellular carcinoma (HCC), liver transplantation history, uncontrolled diabetes mellitus, malignant neoplastic disease, severe cardiac or pulmonary disease, autoimmune disorders, retinopathy, morbid obesity (weight over 125 kg), severe depression, uncontrolled psychotic disorders and existing drug addiction.

The research goals and procedures and the complications of the medications were explained to the patients. Written consents were obtained from all patients. The present study is in full compliance with the ethical principles of the Declaration of Helsinki and the 26 ethical principles mandated by the Iranian Ministry of Health. In addition, an ethical approval was obtained from the National Committee on Medical Ethics, which was registered under IRT138811193307N1 at the Iranian Registry of Clinical Trials (www.irct.ir).

### 3.2. Intervention

A checklist was used to record demographic information; age, gender, HCV risk factor, patients’ weight and height and prior treatment history (naïve, non-responder to previous treatments administered through conventional IFN or relapser to previous treatments). The primary tests included CBC with differential, thyroid function tests (TFTs), liver function tests (LFTs), creatinine and lipid profile. APRI was determined through the following formula:

APRI= [(AST(U/L)/40 (Upper limite normal))×100]/[PLT(/L)/10^9^)]

The HCV genotype was determined through the method explained in reference (19). The HCV RNA was determined using a COBAS Amplicor HCV Monitor, v2.0 (Roche Diagnostics, Branchburg, NJ, USA) with a precision value of 50 IU/ml. The patients underwent intervention treatment taking peginterferon α-2a (Pegasys®, Roche, and Basel, Switzerland) and ribavirin (Copegus®, Roche) in accordance with the standard protocol of the Iranian Ministry of Health. This protocol consisted of, 180µg of peginterferon α-2a administered subcutaneously once per week. In genotype 1 and 4 patients, who weighed less than 75 kg, they were given ribavirin administered orally in 1000 mg daily doses, and in patients who weighed over 75 kg the medication was administered orally in 1200 mg daily doses. In genotype 2 and 3 patients, ribavirin was administered orally in 800 mg daily doses. The treatment length in genotype 1 and 4 patients was 48 weeks and it was 24 weeks in genotype 2 and 3 patients. In addition, patients were followed up six months after the intervention for complications, lab tests and SVR. Patients were monitored through monthly referrals while taking the medications.

### 3.3. Outcome Measurement

The HCV RNA was measured at the outset of the intervention, in weeks 12, 24, 48 and also six months after the end of treatment. The main outcome was SVR levels which were operationally defined as undetectable HCV RNA at the end of the follow-up period. The secondary outcomes included; early viral response (EVR), undetectable HCV RNA or a decrease of more than 2 log10 IU compared to the level at the 12th week of the intervention and the end of treatment response (ETR), undetectable HCV RNA at the end of treatment. On the basis of the obtained outcomes, patients were divided into four groups; non-responder patients with no EVR, breakthrough patients with EVR but without ETR, relapse patients with ETR but without SVR, and patients who had reached SVR. Non-responder patients were considered as treatment failures and their treatment was discontinued.

### 3.4. Assessment of Safety

Lab tests were administered monthly to the patients and any drug complications were addressed through telephone or face-to-face contact both during the intervention and during the follow-up period. The severity of the complications was classified according to WHO categorization as mild, moderate and severe or life threatening. Mild to moderate complications were countered through a decrease in medication dosage or through the prescription of appropriate drugs. Lab criteria for dose reduction of peginterferon α-2a included 500-750/mm^3^ neutrophils or 30000-50000/mm^3^ platelets. The administration of peginterferon α-2a was discontinued in cases of neutrophils less than 500/mm^3^, platelets less than 30000/mm^3^ and hemoglobin levels dropping below 7g/dl (12). In addition, the ribavirin dosage was reduced in cases of hemoglobin levels falling below 10g/dl and it would be stopped if hemoglobin dropped below 8.5g/dl (12). When abnormalities in these lab tests occurred, not only was drug dosage reduced, but the tests also had to be repeated within one or two weeks in order to reach normal levels. The drug dosage was increased again once the tests became normal, otherwise, the treatment would be stopped. G-CSF was prescribed when prolonged dose reduction was required because of neutropenia.

### 3.5. Statistical Analysis

Data were analyzed using SPSS 13.0 for Windows (SPSS Inc., Chicago, IL, USA). The qualitative variables were described using frequency and percentage and the quantitative variables were described using the mean and standard deviation. Data from all patients receiving medication were included in the analysis. Patients reaching SVR were placed in the SVR positive group and the rest of the patients were labeled as the non-SVR group. A chi-square test was used to compare the qualitative variables and an independent sample t-test was performed to compare the quantitative variables. Multivariate logistic regression was performed to evaluate SVR predictors. For this purpose, variables with a P value less than 0.1 in the univariate analysis were considered as predictors of SVR (as the outcome). Variables were entered into the model using the forward conditional with a P = 0.05, and removed with a P = 0.1.

## 4. Results

### 4.1. Patient Characteristics

Seventy-eight percent of the patients were male. The mean age of the patients was 42 years, while 41 patients (41%) were 40 years or younger. A total of 68 patients (68%) had a BMI value of 25 or lower. Intravenous drug user (IVDU) and blood transfusion were the commonest risk factors of HCV. In terms of history, 55 (55%) patients were treatment naïve and the most common virus genotype was 1a. At the outset, an AST or ALT equal to or higher than 40U/L were seen in 64 (64%) and 74 patients (74%), respectively. The AST/ALT was higher than 1 in 27 patients (27%). In addition, 59 patients (59%) demonstrated an HCV RNA level higher than 600 000 IU/ml. Table 1 shows patient characteristics at the outset of the study.

**Table 1:** Basic Characteristics of Patients

**Patient Characteristics**	**Value**
Male, No. (%)	78 (78)
Female, No. (%)	22 (22)
Age, y, mean ± SD	42 ± 12
Age range	17-73
BMI (kg/m^2^), mean ± SD	24.2 ± 3.1
BMI range	18.3-32.7
**HCV risk factors**	
IVDU, No. (%)	33 (33)
Transfusion, No. (%)	39 (39)
Others (surgery, tattoo, etc.) No. (%)	28 (28)
**Genotype**	
1a, No. (%)	70 (70)
1a/3a, No. (%)	4 (4)
1b, No. (%)	10 (10)
3a, No. (%)	15 (15)
Non typeable, No. (%)	1 (1)
**Previous treatment**	
Naïve, No. (%)	55 (55)
Non-Responder, No. (%)	25 (25)
Relapser, No. (%)	20 (20)
WBC (mm^3^), mean ± SD	6027 ± 1859
PMN (mm^3^), mean ± SD	3356 ± 1323
Hb, (g/dl), mean ± SD	14.6 ± 1.8
PLT (mm^3^), mean ± SD	188360 ± 70821
AST, (U/L), mean ± SD	64 ± 51
AST Range	11 - 300
ALT, (U/L), mean ± SD	87 ± 98
ALT Range	10 - 800
HCV RNA, (IU/ml), mean ± SD	1205475 ± 1446704
HCV RNA Range	2040 - 9710000

Abbreviations: ALT, alanine aminotransferase; AST, aspartate aminotransferase; BMI, body mass index; Hb, hemoglobin; HCV, hepatitis C virus; IVDU, intravenous drug user; PLT, platelets; PMN, polymorphoneuclear cells; WBC, white blood cells

### 4.2. Response to Antiviral Therapy

In terms of the ultimate outcomes, 12 patients (12%) (CI95%: 7-20%) were non-responders, 16 patients (16%) (CI95%: 10-24%) were breakthrough, 22 (22%) (CI95%: 15-31%) suffered from a relapse and 50 (50%) (CI95%: 40-60%) had reached SVR. SVR occurred in 31 (56%) of the naïve treatment patients, in 12 (60%) of relapse patients and in seven patients (28%) of non-responder to previous treatment with conventional interferon (IFN). In addition, 88 patients (88%) had EVR with positive SVR occurring in 50 (57%) cases. Table 2 shows antiviral treatment outcomes in patients with a prior treatment history and HCV genotype. Table 3 compares patient characteristics and entry lab test results between SVR and non-SVR groups. As can be seen, SVR mostly occurs in patients who were aged 40 years or lower, who were less likely to be non-responders to previous treatments, who had genotypes other than 1a and who mostly had a baseline HCV RNA of less than 600 000 IU/ml. Predictor variables included significant variables found in the univariate analyses (P < 0.1) and the outcome variables, which also included reaching SVR, were entered into the logistic regression analysis using the forward conditional method. Table 4 shows the results of the multivariate analysis along with the odds ratio (OR) and CI95%. One may infer that an age lower-than-40, a non-1a genotype and an HCV RNA less than 600 000 IU/ml were the best predictors of SVR in decreasing order of strength.

**Table 2:** Treatment Outcome

	**SVR, No. (%)**	**Non SVR, No. (%)**	**Total, No. (%)**
**Previous history**			
Naïve	31 (56)	24 (44)	55 (100)
Relapser	12 (60)	8 (40)	20 (100)
Non-Responder	7 (28)	18 (72)	25 (100)
**HCV Genotype**			
1a	29 (42)	41 (58)	70 (100)
1a/3a	2 (50)	2 (50)	4 (100)
1b	6 (60)	4 (40)	10 (100)
3a	12 (80)	3 (20)	15 (100)
Non-typeable	1 (100)	-	1 (100)

Abbreviation: SVR, sustained virological response

**Table 3:** Univariate Analysis Results Between Patients With and Without Sustained Virological Response (SVR)

	** SVR **	** Non SVR **	** P value **
** Age, y (= 40), No. (%) **	27 (54)	14 (28)	0.008 a
** Sex, (male), No. (%) **	40 (80)	38 (76)	0.629 a
** BMI (kg/m^2^) ( > 25), No. (%) **	18 (36)	14 (28)	0.391 a
** HCV risk factor (IVDU), No. (%) **	15 (30)	18 (36)	0.523 a
** Previous treatment (non-responder) No. (%) **	7 (14)	18 (36)	0.011 a
** Genotype (1a), No. (%) **	29 (41)	41 (57)	0.009 a
** HCV RNA (< 600 000 IU/ml), No. (%) **	25 (50)	16 (32)	0.067 a
** WBC,mm^3^, mean ± SD **	6 164 ± 1 926	5 890 ± 1 820	0.467 b
** PMN,mm^3^, mean ± SD **	3 441 ± 1 297	3 272 ± 1 357	0.527 b
** Hb, g/dl, mean ± SD **	14.8 ± 1.7	14.4 ± 1.8	0.308 b
** PLT ,mm^3^, mean ± SD **	196 020 ± 73 984	180 700 ± 67 382	0.282 b
** AST (= 40 U/L), No. (%) **	34 (68)	30 (60)	0.405 a
** ALT (= 40 U/L), No. (%) **	38 (76)	36 (72)	0.648 a
** AST/ALT > 1, No. (%) **	16 (32)	11 (22)	0.260 a
** APRI = 1.5, No. (%) **	43 (52)	40 (48)	0.424 a
** End of treatment leukopenia c , No. (%) **	26 (52)	25 (50)	0.841 a
** End of treatment neutropenia d , No. (%) **	3 (6)	2 (4)	0.716 a
** End of treatment thrombocytopenia e , No. (%) **	9 (18)	10 (20)	0.761 a

Abbreviations: ALT, alanine aminotransferase; APRI, aspartate aminotransferase (AST) to platelet ratio index; AST, aspartate aminotransferase; BMI, body mass index; Hb, hemoglobin; IVDU, intravenous drug user; PLT, platelets; PMN, polymorphoneuclear cells; WBC, white blood cells

^a^Chi square test

^b^Independent sample t test

^c^End of treatment leukopenia: WBC < 3000/mm3

^d^End of treatment neutropenia: PMN < 750/mm3

^e^End of treatment thrombocytopenia: platelet < 100 000/mm3

**Table 4:** Predictors of Sustained Virological Response (SVR) in Multivariate Analysis

**Predictor**	**Odd’s Ratio**	**CI 95%**
**Age ≤ 40 year**	3.74	1.52 – 9.22
**Genotype non-1a**	3.71	1.40 – 9.81
**HCV RNA < 600 000 IU/ml**	2.52	1.03 – 6.15

Nagelkerke R-square = 0.232

### 4.3. Drugs Dosage and Complications

Drug dosage for 14 patients (14%) was reduced during the study (ribavirin in five patients, peginterferon in seven patients, and both medications in two patients). The treatment of two patients was terminated at the ninth month, one due to intolerance of the medication and another because of occurrence of lichen planus. Mortality and loss at follow-up was zero. Hemoglobin levels never dropped below 7g/dl in any of the patients involved in this study. At the end of the treatment, 51 patients (51%) suffered from leukopenia, five (5%) from neutropenia and 19 (19%) from thrombocytopenia. No major abnormalities were seen in; liver, kidney and thyroid tests and/or lipid profiles. Table 5 shows the prevalence of drug complications in patients.

**Table 5:** The Prevalence of Drug Complications

	**No. (%)**
**Fatigue**	74 (74)
**Myalgia**	68 (68)
**Depression**	39 (39)
**Headache**	38 (38)
**Flu like**	37 (37)
**Fever**	31 (31)
**Itching**	25 (25)
**GI symptoms**	21 (21)
**Hair loss**	16 (16)
**Chills**	10 (10)
**Rash**	8 (8)
**Insomnia**	7 (7)
**Dry skin**	7 (7)

## 5. Discussion

The findings of the present study show that half of the patients receiving a combined peginterferon α-2a and ribavirin treatment reached SVR, and these rates were different among the various genotypes. The highest SVR was observed in patients with genotype 3a, while the lowest SVR was identified in patients with genotype 1a. In addition, age, virus genotype and the baseline viral load, proved to be strong SVR predictors. In fact, an age less than 40 years, non-1a genotypes and viral loads of less than 600 000 IU/ml correlated with a higher SVR. However, our study did not show end-of-the-treatment; leukopenia, neutropenia, thrombocytopenia, and also the APRI as predictors of reaching SVR. Evidence shows that the majority of patients with SVR sustain negative HCV RNA for years. Such patients indicate a reduced necroinflammatory process and a slight reduction in the reversible components of fibrosis. In addition, such patients show appropriate long-term biochemical and histological outcomes (3). Furthermore, SVR can lead to improved quality of life (24). Therefore, reaching SVR remains an important goal for HCV patients.

Studies in the past decade have showed that using peginterferon instead of interferon in combination with ribavirin in the treatment of HCV patients leads to improved treatment outcomes in these patients with ETR and SVR rates increasing to 69% and 56%, respectively, in patients with different genotypes (9). In addition, peginterferon is shown to have increased SVR in patients with genotype 1 to 52% and to 80% in patients with genotypes 2 or 3 (25, 26). Nevertheless, mixed treatment results have been observed in different patient populations. In a recent study, McHutchinson et al. reported an SVR of 40.9% in 3070 patients with genotype 1 (27). Rumi et al. observed an SVR of 66% (among 431 patients with genotypes 1, 2, 3 and 4) (28). Ascione et al. administered a combined peginterferon α-2b and ribavirin treatment to 320 patients with genotypes 1, 2, 3 and 4 and reported an SVR of 68.8% (29). In a retrospective study on 2378 patients, Mauss et al. reported an SVR of 57.9% in patients with genotypes 1 and 4 and an SVR of 77.3% in patients with genotypes 2 and 3 (30). Although our study showed an SVR of 50% in patients, this difference could be accounted for by differences in patient genotypes and their treatment history.

Nevertheless, our findings are to some extent, in line, with those of previous studies conducted locally. One example is Alavian’s study on 52 patients that showed an SVR of 53.8%, although their genotypes were not included as a possible variable (18). In addition, in that study, response to treatment among naïve patients was reported to be 62.9% (18), while our study records a lower level of 56%. Zali et al. conducted a study on 57 patients (some suffering from thalassemia or hemophilia) and reported an SVR of 50% in all patients and 66.7% in patients with a prior treatment history (23). In a similar study in 2004, Daryani et al. reported an SVR of 78.3% in 23 patients (21). Although their result is significantly higher than similar studies in Iran and in other countries, it could be justified by the exclusion of patient genotypes and the small number of patients with a prior history of treatment. Bafandeh et al. in 2007 reported an SVR of 48% in 118 patients (20). In a study on hemophilic patients, Alavian et al. reported an SVR of 61% (12) and finally in a study on 216 patients in Tehran, Jabari et al. recorded an SVR of 77.8% (22) which holds a higher result to that of ours. However, one should note that in their study all patients were treatment naïve and almost half of them had genotypes 2 or 3. In light of the results from the foregoing studies, one may state that the SVR rate in different populations, even in the same geographical location, depends on patient characteristics and viral properties. For instance, the SVR rate in American patients is less than European ones (15, 16, 31, 32) while that of Asian patients outscores both (33). In general, reaching an SVR in patients with a prior history of treatment has been reported to be 20-30% (34). In the study by Alavian et al., SVR in naïve patients was found to be 62.9% and in non-responder or relapsing patients it was found to be 35.3% (18). Our study showed an SVR proportion of 56%, 60% and 28% in naïve, relapse and non-responder patients using conventional interferon, respectively. These results show that combination therapy with peginterferon α-2a and ribavirin can be effective for a third of patients with a history of treatment. Our study also showed that the most significant predictors of SVR were; an age below 40, non-1a genotype and a viral load less than 600 000 IU/ml. Numerous variables have been recognized to affect a patient’s response to antiviral treatment, including; age, genotype, viral load, patient weight, race and environmental factors, etc. (12, 15, 16, 31, 32). Genotype has been the most important predictors in a variety of studies (11, 13, 35). In comparison to other genotypes, genotype 1 has been associated with lower SVR (31, 32, 36). Genotypes other than 1a have been shown to have independently 3.25-5.4 times higher chance of positive response to treatments in various clinical trials (36). The SVR rate in genotype 1 patients increased from 41% to 52% after 48 weeks of treatment (9, 36) while a similar regimen produced an SVR of 76%-84% in genotypes 2 and 3 in a treatment period of 24-48 weeks (9, 36, 37). Our study also showed that the chances of reaching SVR in patients with a non-1a genotype are 3.71 times higher than those of 1a genotype patients. In addition, a low viral load can be an independent predictor for SVR (12, 17, 31, 36), overshadowing basic patient characteristics as predictors for reaching SVR (38). In our study, the chances of patients with viral load levels less than 600 000 IU/ml reaching SVR were 2.52 times higher.

Another predictor factor in reaching SVR is an age of 40 to 45 or lower (30, 39) which increases the chances of reaching SVR by 3.74 times. Although other studies have presented variables such as; gender, higher weight or BMI, ETR leukopenia, thrombocytopenia and neutropenia as SVR predictors (12, 36, 40), our study did not prove these variables to be predictors. In addition, although APRI is used as an index to rate liver fibrosis (41), it failed to function as an SVR predictor in our study. In general, although different studies have introduced different SVR predictors (which may be due to different sample sizes, patients or virus characteristics), the majority of previous studies emphasize virus characteristics (ie, virus genotype or viral load) as the primary predictors of SVR in comparison with patient characteristics. Given the numerous complications, lengthy treatment period and high medical costs, it seems that determining and using SVR predictors at the outset of the HCV treatment may be beneficial (42). Hence, numerous studies are under way to find response-to-treatment predictors. An example would be detecting genetic variations of the IL28B gene in chromosome 19, which seems to be related to response-to-treatment in patients with genotype 1 (43). Although our study was one of the few studies in Iran conducted on HCV patients with advantages in terms of sample size and the multiplicity of SVR predictors, it should be noted that since patients in clinical trials were selected by exact inclusion criteria for entry into the study (selection bias), the results of these studies may not be generalized to the patient population in terms of SVR measure or SVR predictors. As a matter of fact, some studies have shown a lower SVR in general populations compared to clinical trial samples (42, 44). Therefore, despite improvements in antiviral therapy in recent years, the treatment of chronic hepatitis C is still a challenging endeavor requiring significant improvement (17). Our findings show that achieving SVR through a combination therapy with peginterferon α-2a and ribavirin in chronic HCV patients is 50%. The highest SVR rate was reported in genotype 3a patients (80%) and the lowest rate was observed in genotype 1a patients (42%). In addition, age, virus genotype and viral load at the outset of the treatment emerged as strong predictors of SVR.

Achieving SVR slows the progress of the disease and it is a significant treatment goal. Due to numerous medication complications, lengthy treatment period and high medical costs associated with the treatment of these patients, further research is strongly recommended in order to investigate other likely SVR predictors and to find new drugs that may increase SVR rates in different patient populations.
